# Localization of VEGF to Vascular ECM Is an Important Aspect of Tumor Angiogenesis

**DOI:** 10.3390/cancers9080097

**Published:** 2017-07-28

**Authors:** Weon-Kyoo You, William B. Stallcup

**Affiliations:** 1 ABL Bio Inc., Gyeonggi-Do 13488, Korea; weonkyoo.you@ablbio.com; 2 Tumor Microenvironment and Cancer Immunology Program, Cancer Center, Sanford Burnham Prebys Medical Discovery Institute, La Jolla, CA 92037, USA

**Keywords:** VEGF, vascular basement membrane, tumor vascularization, vessel diameter, vascular function, hypoxia, pericytes, endothelial cells, macrophages, invadopodia

## Abstract

Our research has identified several examples in which reduced VEGF-A binding to deficient vascular extracellular matrix leads to deficits in tumor vascularization and tumor growth: (1) germline ablation of collagen VI in the stroma of intracranial B16F10 melanomas; (2) knockdown of the Tks5 scaffolding protein in MDA-MB-231 mammary tumor cells; (3) germline ablation of NG2 proteoglycan in the stroma of MMTV-PyMT mammary tumors; and (4) myeloid-specific ablation of NG2 in the stroma of intracranial B16F10 melanomas. Tumor hypoxia is increased in each of the four types of experimental mice, accompanied by increases in total VEGF-A. However, while VEGF-A is highly associated with tumor blood vessels in control mice, it is much more diffusely distributed in tumors in all four sets of experimental mice, likely due to reduced extent of the vascular extracellular matrix. In parallel to lost VEGF-A localization, tumor vessels in each case have smaller diameters and are leakier than tumor vessels in control mice. Tumor growth is decreased as a result of this poor vascular function. The fact that the observed vascular changes occur in the absence of alterations in vascular density suggests that examination of vessel structure and function is more useful than vascular density for understanding the importance of angiogenesis in tumor progression.

## 1. Introduction

Microvessels are primarily composed of three elements: endothelial cells, pericytes, and the vascular basement membrane. While endothelial cells receive an outsized share of attention in research on microvessels, the importance of pericytes and the basement membrane should nevertheless not be understated. In point of fact, the three microvascular components are not independent entities, but instead interact in complex ways to determine vessel development, maturation, maintenance, and function [1,2,3,4,5]. These studies from our lab and others make it clear that assembly of the vascular basement membrane depends on cooperation between pericytes and endothelial cells, and that the properties of the two vascular cell populations rely in turn on their interaction with the basement membrane.

Vascular endothelial growth factor (VEGF) is widely recognized in the literature as a key agent in controlling endothelial cell biology and in promoting angiogenesis [6,7,8,9]. The role of VEGF in tumor angiogenesis is especially well-studied, and anti-angiogenic therapy via inhibition of VEGF signaling can be an effective means of interfering with tumor growth [9,10,11,12]. A subset of VEGF-related research has also established that the localization/sequestration of the growth factor to specific sites is important for its actions on the vasculature. In particular, binding of VEGF to extracellular matrix components appears to be a key aspect of the factor’s action [13,14,15,16]. Our laboratory has reported several examples in which reduced VEGF-A binding to deficient vascular extracellular matrix leads to deficits in tumor vascularization. In these examples, different mechanisms are responsible for aberrant assembly of the vascular extracellular matrix and for associated alterations in the properties of pericyte and endothelial cells. Assembly of the vascular basement membrane is rather directly affected in some of these cases, while in others the effect is more indirect. However, regardless of differences in mechanism, the loss of VEGF-A localization to the vascular basement membrane is seen in all four cases examined here, leading in each case to deficits in tumor vascularization and diminished tumor growth.

## 2. Direct Effects on the Vascular Basement Membrane Alter VEGF-A Localization and Tumor Vascularization

In two examples, we have seen changes in tumor vascularization and growth that appear to result from relatively direct effects on assembly and processing of the vascular extracellular matrix.

### 2.1. Collagen VI Ablation in Host Stroma of Intracranial Melanomas

Collagen VI is a rather unique collagen species that provides a link between cell surface receptors, such as integrins [17] and the nerve-glia antigen-2 (NG2) proteoglycan [18], and fibrillar collagens such as collagens I and IV that are involved in basement membrane assembly [19,20]. Thus, collagen VI can be regarded as a focal point for deposition of the vascular basement membrane. Indeed, an early study from our lab on brain tumor vascularization revealed that genetic ablation of collagen VI was detrimental to assembly of the collagen IV-rich vascular basement membrane [21]. This initial result suggested the potential utility of the collagen VI null mouse [22] for more detailed examination of the effects of basement membrane deficits on tumor vessel development and function, and ultimately on tumor growth. We investigated this topic by studying the vascularization of intracranial melanoma tumors established by microinjection of B16F10 cells [23] into the brains of wild type and collagen VI null C57BL/6 mice [24]. Growth of the tumors served as an overall readout for vascular function, and more detailed histological studies were used to establish the detailed structural and functional properties of tumor vessels in the two lines of mice.

Tumor volumes in collagen VI null mice were roughly half the volumes seen in wild type mice at 12 and 15 days post-engraftment (Table 1). Since the tumor cells were identical in both mouse lines, differences in the host microenvironment must explain this alteration in tumor growth. Since the tumor vasculature is a prominent host-derived component of the microenvironment, we first compared the most overt properties of tumor vessels in wild type and collagen VI null hosts. While microvascular density was similar in tumors in both mouse lines, vessel diameter was reduced in the case of collagen VI null hosts (Table 1). This 30% decrease in vessel diameter translates into roughly a 70% reduction in the volume of blood carried by tumor vessels in collagen VI null mice, providing a reasonable explanation for the diminished tumor growth seen in these mice. Tumor vessels in collagen VI null mice were also characterized by a decrease in the width of the vascular basement membrane [24]. The extent of this decrease was estimated at between 30% to 50% of the width of the basement membrane in wild type mice (Table 1), depending on whether we used immunolabeling for collagen IV, collagen I, or laminin to mark the extent of the structure.

These changes in the vascular basement membrane in collagen VI null hosts were not accompanied by changes in the number of pericytes or endothelial cells associated with tumor vessels, or by changes in the extent of pericyte ensheathment of endothelial cells [24]. However, we were able to detect changes in the properties of both vascular cell types as a result of basement membrane alteration in the absence of collagen VI. Pericyte maturation was diminished, as judged by expression of α-smooth muscle actin (Table 1). In the case of endothelial cells, apoptosis was increased, while the number of sprouting tip cells was decreased (Table 1).

**Figure 1:**
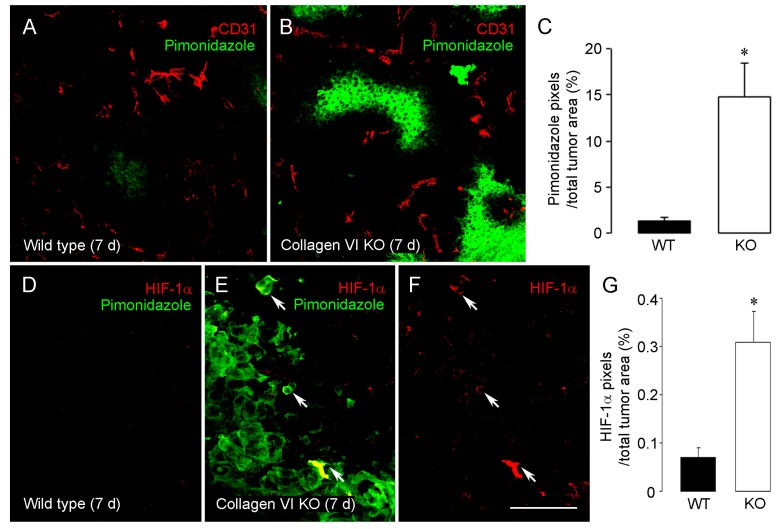
Increased hypoxia and HIF1α expression in tumors in collagen VI null mice. Hypoxia levels in 7-day intracranial B16F10 tumors were determined after intravenous injection of a pimonidazole hypoxia probe (60 mg/kg, 1 h circulation period). Tumor sections were double-stained for pimonidazole (green) and CD31 (red). In contrast to rare pimonidazole-positive regions in wild type tumors (**A**), areas of intratumoral hypoxia were markedly increased in tumors from collagen VI null mice (**B**). Intratumoral hypoxia levels, defined as the percentage of total tumor area covered by pimonidazole pixels, were increased 10-fold in collagen VI null mice (**C**). Very low levels of immunolabeling for HIF-1α (red) were detected in tumors in wild type mice (**D**), in agreement with the virtual absence of immunolabeling for pimonidazole (green). In tumors in collagen VI null mice, increased hypoxia detected via pimonidazole labeling was accompanied by more abundant labeling for HIF-1α ((**E**) and (**F**)). HIF-1α levels are more than 3-fold higher in collagen VI null mice than in wild types (**G**) * *p* < 0.05 vs. wild type. Scale bar: 120 μm in (**A**) and (**B**); 40 μm in (**D**–**F**). Data taken from (You et al. [24]).

As a result of these cellular and structural changes, tumor vessels in collagen VI null hosts exhibited decreased patency (as measured by perfusion with FITC-labeled LEA lectin) and increased leakiness (as measured by leakage of FITC-dextran) (Table 1) [24]. These factors likely couple with reduced vessel diameter to reduce even further the ability of tumor vessels to nourish tumors in collagen VI null hosts, as reflected by a large increase in tumor hypoxia in the null mice (Figure 1A–C; Table 1). As expected, levels of HIF-1α were also increased in these hypoxic collagen VI null tumors, compared to tumors in wild type mice (Figure 1D–G; Table 1).

**Figure 2:**
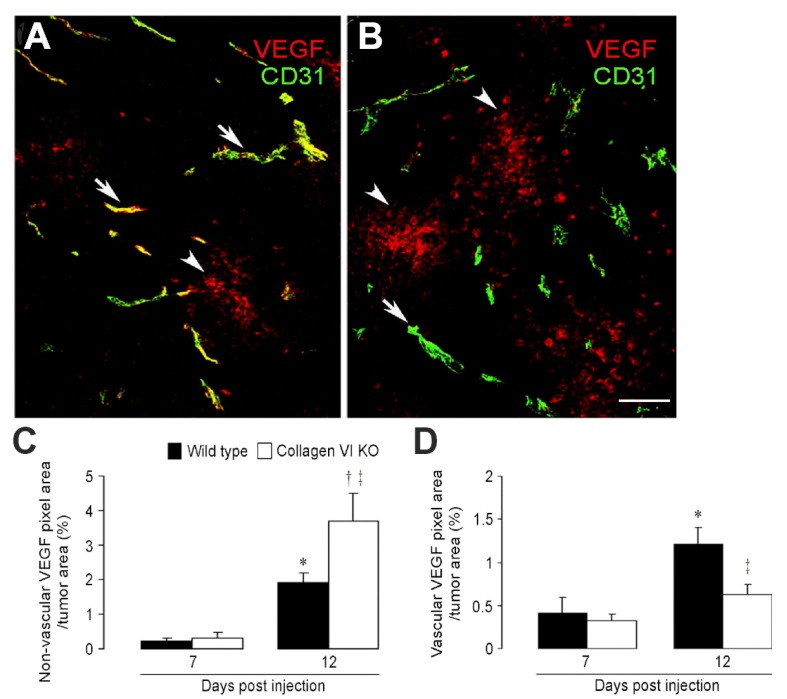
Altered VEGF-A localization following ablation of collagen, V.I. Immunostaining for VEGF-A (red) and CD31 (green) was used to quantify and localize VEGF-A sequestration in tumors from control (**A**) and collagen VI null (**B**) mice. Arrows identify VEGF-A closely associated with tumor blood vessels. Arrowheads identify VEGF-A distributed more diffusely in the tumor parenchyma. Non-vascular VEGF-A in collagen VI null tumors is increased 2-fold compared to control tumors (**C**). In contrast, vascular VEGF-A in collagen VI null tumors is reduced 2-fold compared to control tumors (**D**). Scale bar: 120 μm. * *p* < 0.05. † *p* < 0.05 versus 12-day wild type. ‡ *p* < 0.002 versus 7-day collagen VI null. Data taken from You et al. [24].

While increased levels of HIF-1α led to the expected upregulation of VEGF-A expression in both sets of tumors at 12 days post-engraftment, the details of VEGF-A localization differed in wild type and collagen VI null hosts [24]. In tumors in wild type hosts, the majority of VEGF-A was closely associated with tumor vessels (Figure 2A). In tumors in collagen VI null hosts, most VEGF-A was not associated with tumor vessels, but was distributed more diffusely in the tumor stroma (Figure 2B). Figure 2C,D quantify the percentages of vascular VEGF-A versus non-vascular VEGF-A in the two sets of tumors. In light of the observed ability of extracellular matrix components to sequester VEGF, it seems plausible that the decrease in vessel-associated VEGF-A in collagen VI null tumors was due to the reduced extent of the vascular basement membrane in these mice. This idea will be further explored in the following sections.

### 2.2. Tks5 Knockdown in Mammary Tumor Cells

The Tks5 Src substrate is a novel scaffolding protein thought to be essential for the formation and function of invadopodia [25,26], actin-rich structures thought to contribute to the ability of tumor cells to penetrate and remodel the extracellular matrix [27,28]. Since high levels of Tks5 expression were found to be correlated with poor patient outcome in cases of invasive stage I and II breast cancer, the effects of Tks5 knockdown were investigated on orthotopic growth of MDA-MB-231 human breast cancer cells in SCID-Beige mice [29]. Table 1 shows that there was a large decrease in tumor growth in the case of Tks5 knockdown cells.

**Figure 3:**
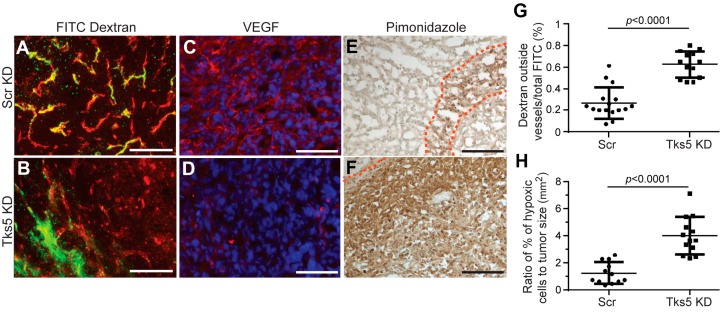
Reduced Tks5 expression in mammary tumor cells results alters tumor blood vessels. Blood vessels in mammary tumors formed by MDA-MB-231 cells transfected with scrambled shRNA (Scr KD) and Tks5 shRNA (Tks5 KD) were analyzed for leakage of FITC-dextran, localization of VEGF-A, and levels of tumor hypoxia. (**A**) and (**B**): Levels of FITC-dextran (green) outside of CD31-positive vessels (red) were determined by confocal microscopy. Increased leakage from vessels in Tks5 knockdown tumors is quantified in (**G**). (**C**) and (**D**): Confocal microscopy of immunolabeling sections was used to localize VEGF-A (red) to tumor vessels in control tumors. This vascular localization is largely lost in Tks5 knockdown tumors. Blue = DAPI. (**E**) and (**F**): Pimonidazole was injected intravenously into tumor-bearing mice and allowed to circulate for 10 min. Immunolabeling was then used to localize and quantify pimonidazole accumulation in areas of hypoxia. Dashed lines delineate the borders of hypoxic areas. Hypoxia is greatly increased in Tks5 knockdown tumors (**H**). Scale bars: 100 μm. Data taken from Blouw et al. [29].

Since we were not initially anticipating tumor cell-dependent changes in vascularization in this study, many of the parameters of tumor vessel structure examined in our collagen VI study were not examined in the Tks5 knockdown model. However, a large decrease in vessel diameter was noted (Table 1), indicating that vascular deficiency could provide at least part of the explanation for reduced breast cancer growth. This idea was supported by finding that vessels in the Tks5 knockdown tumors were leakier than in control tumors and that levels of tumor hypoxia were also elevated (Figure 3; Table 1). As in tumors in collagen VI null mice, tumor vessel density was unchanged in Tks5 knockdown tumors (Table 1). Interestingly, double labeling for CD31 and VEGF-A revealed that VEGF-A association with vessels in Tks5 knockdown tumors was much lower than in control tumors (Figure 3; Table 1), suggesting the same type of correlation between reduced vascular VEGF-A and vessel diameter and function that we noted in tumors in collagen VI null mice. To complete the parallel between the two models, it remains to be determined whether alterations in the vascular basement membrane in Tks5 knockdown tumors are responsible for the loss of VEGF-A association with the vasculature. However, the association of Tks5 with invadopodia, coupled with the matrix altering functions of invadopodia, are consistent with the idea that changes in the basement membrane could underlie the observed changes in VEGF-A localization. Alternatively, altered proteolytic processing of VEGF-A due to Tks5/invadopdia loss might provide a mechanism for changing VEGF-A localization/availability.

## 3. Indirect Effects on the Vascular Basement Membrane Alter VEGF-A Localization and Tumor Vascularization

Because endothelial cells and pericytes cooperate in the deposition of the vascular basement membrane [30], deficits in either of these two cell types or in their interaction can indirectly affect the composition and/or extent of the basement membrane. We have encountered two interesting examples of this phenomenon: (a) in the case of MMTV-PyMT mammary tumors grown orthotopically in germline NG2 proteoglycan null mice [31] and (b) in the case of B16F10 melanomas grown intracranially in mice in which NG2 is specifically ablated in myeloid cells [32].

### 3.1. Germline NG2 Ablation MMTV-PyMT Mammary Tumor Stroma

Mammary tumor virus promoter-driven expression of the polyoma middle T oncogene (MMTV-PyMT) is a widely used model for studying mammary tumorigenesis [33,34]. Although the NG2 proteoglycan is not expressed by mammary tumor cells in the MMTV-PyMT model, it is expressed by pericytes and macrophages in the tumor microenvironment. Germline ablation of NG2 slowed the progression of both spontaneous and engrafted mammary tumors in this model (Table 1), demonstrating the power of the tumor stroma in promoting tumor growth [31]. While there was no change in tumor vessel density, the diameter of blood vessels in these NG2 null mammary tumors was diminished, and several changes in vessel structure and function could be quantified (Table 1). Unlike the situation in collagen VI null tumors in which we observed no changes in pericyte-endothelial cell interaction, pericyte ensheathment of vascular endothelial cells in NG2 null mammary tumors was reduced, affecting the biology of both cell types, as well as assembly of the vascular basement membrane. Pericyte maturation and endothelial cell sprouting were both impaired, and the extent of the vascular basement membrane was decreased (Table 1). As will be further described in the following section, these phenomena are the combined result of ablating NG2 in both pericytes and macrophages, since both types of NG2 loss affect pericyte-endothelial cell interaction.

**Figure 4:**
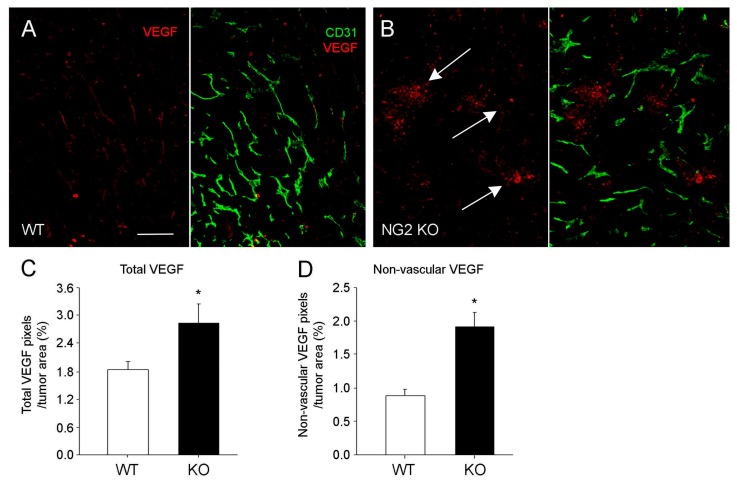
Localization of VEGF-A expression in mammary tumors. Double immunolabeling for CD31 (green) and VEGF-A (red) was used to localize VEGF-A expression relative to mammary tumor blood vessels in control (**A**) and NG2 null (**B**) mice. Total VEGF-A is elevated in tumors in NG2 null mice (**C**), but this increase is due to an increase in non-vascular VEGF-A (**D**). In tumors in control mice, VEGF-A expression is largely associated with the vasculature, while more diffusely localized VEGF-A is evident in tumors in NG2 null mice (arrows in B). * *p* < 0.01. Scale bar: 60 μm. Data taken from Gibby, et al. [31].

As a result of these structural changes in mammary tumor blood vessels, vessel functionality was also impaired, as evidenced by decreased patency and increased leakiness (Table 1). Accordingly, tumor hypoxia increased, along with overall levels of VEGF-A. However, much of the increased VEGF-A was diffusely localized in the tumor rather than associated with tumor blood vessels (Table 1; Figure 4B), a pattern we have already seen in the examples of tumors in collagen VI null mice and Tks5-knockdown mammary tumors. This reinforces the link between diminished vascular extracellular matrix (ECM), VEGF-A localization, deficits in vessel structure and function (including vessel diameter), and tumor growth.

### 3.2. Myeloid-Specific NG2 Ablation in Host Stroma of Intracranial Melanomas

Myeloid-specific NG2 null (Mac-NG2ko) mice were generated by crossing NG2 floxed mice [35] with LysM-Cre transgenic mice [36,37]. NG2 null macrophages in these mice exhibit greatly reduced recruitment to both B16F10 brain tumors and sites of spinal cord demyelination [32,38,39].

**Figure 5:**
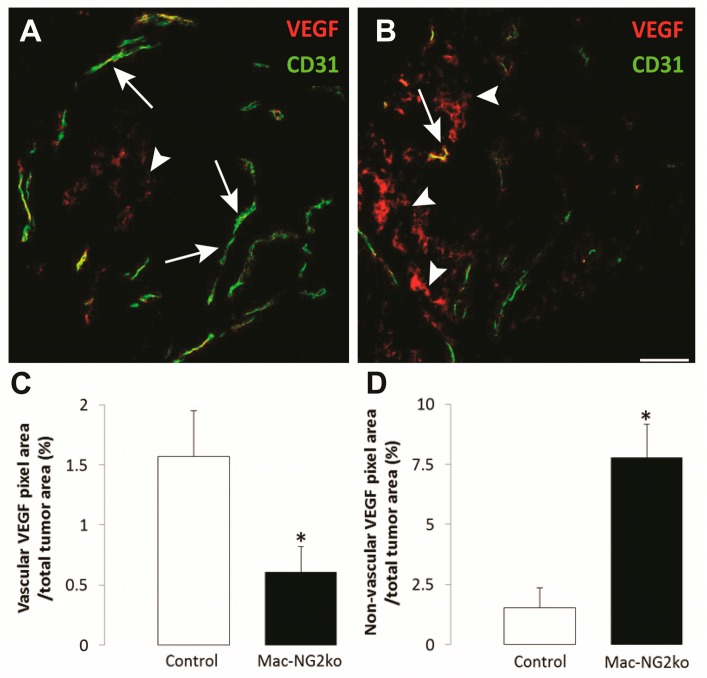
Altered VEGF-A localization following myeloid-specific ablation of NG2. Double immunostaining for VEGF-A (red) and CD31 (green) was used to quantify and localize VEGF-A sequestration in tumors from control (**A**) and Mac-NG2ko (**B**) mice. VEGF-A in control tumors is highly localized to blood vessels, while vascular VEGF-A in Mac-NG2ko tumors is reduced 3-fold (**C**). Instead, non-vascular VEGF-A in Mac-NG2ko tumors is increased by a factor of 5 (**D**). Scale bar = 60 μm. * *p* < 0.01 compared to controls. Data taken from Yotsumoto, et al. [32].

Macrophages are increasingly recognized as promoters of tumor angiogenesis, in part because they are a rich source of factors that influence the biology of vascular cells [40,41,42,43]. In the case of our studies on intracranial melanomas in Mac-NG2ko mice, the decreased number of tumor macrophages led to diminished ability of pericytes to interact with endothelial cells [32]. This is likely due to loss of a macrophage-derived signal normally required to stimulate expression of molecules such as N-cadherin [1,44,45] that participate in pericyte-endothelial cell recognition. Further work is required to identify the mechanism(s) by which NG2 enhances macrophage recruitment and to determine if the loss of NG2 expression affects the recruitment of specific macrophage populations (for example, based on M1/M2 polarization status). Nevertheless, this loss of pericyte-endothelial recognition in tumors in Mac-NG2ko mice resulted in greatly diminished pericyte ensheathment of endothelial cells (Table 1). This loss of interaction had severe consequences for both vascular cell types and for the structure and function of tumor vessels in the Mac-NG2ko mice. Pericyte maturation was reduced, while endothelial cell sprouting was decreased (Table 1) and endothelial junction formation was diminished by a factor of 2 [32]. Interestingly, these deficits in Mac-NG2ko mice were larger than those seen in vessels in tumors in pericyte-specific NG2 null mice [32,46], in which loss of NG2 by pericytes also led to reduced pericyte ensheathment of endothelial cells (Figure 5A,B). Ablation of NG2 in pericytes did not result in the loss of pericyte ability to recognize endothelial cells (as in Mac-NG2ko mice), but only in the strength of the pericyte-endothelial interaction, based on loss of NG2-stimulated β1 integrin signaling in the endothelial cells [46,47]. Paralleling the severity of these changes in pericyte-endothelial cell interaction, intracranial tumor growth was also more severely curtailed in Mac-NG2ko mice than in pericyte-specific NG2 null mice, although in both cases tumor growth was slower than in control mice [32].

Importantly, basement membrane assembly was also decreased in tumors in Mac-NG2ko mice by the loss of pericyte-endothelial cell interaction, so that the association of endothelial cells with collagen IV in the vascular basement membrane was greatly reduced (Table 1). Strikingly, tumor vessel diameter was decreased by 50% in tumors in Mac-NG2ko mice (Table 1), reminiscent of the reduced tumor vessel diameter seen in each of the three previous examples. In contrast, tumor vessel diameter was not reduced in pericyte-specific NG2 null mice, apparently due to the less severe deficit in pericyte-endothelial cell interactions and in accompanying changes that occur in pericyte and endothelial cell biology. Vessel function in Mac-NG2ko tumors was further compromised by a decrease in vessel patency and by an increase in vessel leakiness, resulting in a large increase in tumor hypoxia (Table 1) and a 2-fold rise in HIF-1α expression. These negative trends in vessel structure and function in tumors in Mac-NG2ko mice [32] are all similar to those observed in tumor vessels in the three other examples discussed here.

As expected, increased HIF-1α expression in response to hypoxia in tumors in Mac-NG2ko mice was accompanied by increases in VEGF-A levels. Moreover, the localization of VEGF-A expression in Mac-NG2ko mice differed from that seen in control mice (Figure 5A,B). Levels of VEGF-A associated with tumor vessels in Mac-NG2ko mice were several-fold lower than in vessels in control mice (Figure 5C). Conversely, levels of diffusely-distributed non-vascular VEGF-A in tumors in Mac-NG2ko mice were much greater than in control mice (Figure 5D). These observations reinforce the concepts developed in the case of the collagen VI null mouse, the NG2 null mouse, and the Tks5 knockdown mammary tumors; namely, that reduced deposition of the vascular basement membrane results in loss of sequestration of VEGF-A in close proximity to tumor vessels, with negative consequences for vessel structure and function, as well as for tumor growth. It is important to note that the observed changes in vessel structure and function occurred in the absence of changes in tumor vessel density, a phenomenon that was also seen in each of the other three tumor models. This suggests that examination of tumor vessel structure and function can be more valuable than vessel density in understanding the role of the vasculature in tumor progression.

## 4. Discussion

Using both intracranial and mammary tumors, our lab has identified several examples in which diminished assembly of the vascular basement membrane is linked to additional deficits in the structure and function of tumor blood vessels. The results of these studies highlight the intimate interplay between endothelial cells, pericytes, and the vascular extracellular matrix and demonstrate the extent to which deficits in one of these compartments leads to deficits in the other compartments. Due to the inability of deficient vessels to support robust tumor growth, these vascular deficits are associated with decreased tumor progression. It is important to emphasize that in each of the four cases presented here we have studied the properties of tumor blood vessels within 7–10 days of tumor initiation when the tumors had reached a diameter of only a few millimeters. As frequently noted in other reports, vessels often become chaotic and tortuous at later stages of tumor development, a phenomenon that interfered with our ability to obtain statistically significant data for several of the quantitative assessments of vessel structure and function presented in our studies. Our conclusions regarding the effects of perivascular VEGF-A therefore only apply to the early stages of tumor development when vessels exhibit relatively “normal”, non-chaotic morphologies. At this early stage, the effects of perivascular VEGF-A localization might be interpreted as contributing to “vessel normalization”, a process in which the structure and function of vessels are optimized for efficient delivery of blood [48,49]. Perivascular VEGF-A localization appears to contribute to vessel normalization in the sense that vessel structure and function in tumors in control mice are superior to the structure and function of tumor vessels in the various gene ablation models.

In one of our experimental examples, genetic ablation of the linker protein collagen VI leads directly to alterations in the assembly of the collagen matrix that is a key part of the vascular basement membrane. This extracellular matrix deficit leads to deficiencies in the development of both pericytes and endothelial cells, and thus to changes in vascular structure that adversely affect vessel function. In a second example, Tks5 ablation in mammary tumor cells leads to putative alterations in matrix processing, once again with negative effects on vessel function. In a third example, germline ablation of the NG2 proteoglycan diminishes pericyte interaction with endothelial cells, leading indirectly to altered assembly of the vascular basement membrane. In a fourth example, ablation of NG2 specifically in myeloid cells results in reduced recruitment of tumor macrophages, a deficit that leads indirectly to alterations in basement membrane assembly based on the inability of pericytes to recognize and interact with endothelial cells to promote extracellular matrix deposition. These latter two examples illustrate how reduced pericyte-endothelial cell interaction can negatively affect assembly of the vascular matrix, resulting in poor vessel function. Despite these differences in underlying mechanisms, all these cases of diminished basement membrane assembly are associated with a diffuse pattern of VEGF-A localization in tumors that contrasts sharply with the highly perivascular localization/sequestration of VEGF-A observed in tumors in control mice.

The literature on human brain tumors offers precedents for perivascular localization of VEGF. For example, strong immunolocalization of VEGF to tumor blood vessels, without apparent VEGF expression in tumor cells, has been reported in human oligodendrogliomas [50,51]. In contrast, other workers have reported VEGF expression in both of these compartments [52]. While it is possible that these different results stem from differences in antibody recognition of VEGF isoforms, it is also possible that the results reflect real differences in VEGF localization in different tumors.

Based on a growing literature detailing the functional importance of VEGF interaction with extracellular matrix components, our hypothesis is that the reduced extent and/or altered composition of the vascular basement membrane in each of our experimental models is responsible for this loss of perivascular VEGF-A sequestration. An interesting parallel from a completely different system is instructive. Loss of heparin sulfate results in an abnormally diffuse distribution of BMP2 in the developing limb and thus to changes in the spatial localization of BMP2 signaling [53]. This leads to skeletal defects, much as our observed changes in VEGF-A localization lead to vascular defects. It will be important in future work to determine if deficits in VEGF-A sequestration/localization can be linked to the loss of specific extracellular matrix components or whether the deficiency is simply due to the reduced extent of the extracellular matrix.

The effects of VEGF on endothelial cells and vascularization depend on several factors, including the alternative splicing of VEGF messages, the source of the VEGF, VEGF processing, the level of VEGF expression, and the sequestration/localization of VEGF in the vascular basement membrane [13,54]. The observations from our studies are most relevant to the latter two issues. Our results confirmed that increased tumor hypoxia and the resulting elevation of HIF-1α expression in our experimental mice were associated with increased VEGF-A expression, an outcome consistent with the extensive literature on this topic [55,56,57]. However, the elevated VEGF-A expression in tumors in our experimental mice did not result in improved vascular function in these tumors, a consequence that appeared to coincide with the reduced sequestration of VEGF-A in perivascular sites. The basement membrane contains several different species of extracellular matrix molecules, including collagens, laminins, fibronectin, and proteoglycans [2,4]. In addition to providing structural support for the basement membrane and vasculature, these components also have important effects on vascular cell biology. For example, given that fibronectin [58,59,60,61] and proteoglycans [16,62,63] are key players in mediating/controlling VEGF-A binding, it is not surprising that deficits in basement membrane assembly can have significant effects on VEGF-A localization. The heparan sulfate proteoglycan perlecan is able not only to sequester VEGF, but also to enhance VEGF presentation to VEGF receptors as a means of potentiating responses in endothelial cells [16,62]. Similarly, binding of heparin to a specific domain of fibronectin promotes fibronectin sequestration of VEGF and subsequent promotion of VEGF interaction with VEGF receptors [59,61,64]. Moreover, loss of integrin binding sites in the basement membrane, due to diminished deposition of collagens, laminins, and fibronectin, has additional negative effects on vascular cell adhesion, growth, and survival [65,66]. The importance of integrin signaling has been especially well-documented in the case of endothelial cells [14,60,65,66]. Loss of integrin-mediated signaling in endothelial cells couples with the loss of spatial cues normally provided by perivascular VEGF-A localization to undermine the development and maintenance of blood vessels.

An important observation from our studies is that the association of a robust vascular basement membrane with tumor vasculature in wild type mice did not support superior tumor vascularization by promoting the development of larger numbers of blood vessels. On the contrary, microvascular density was unchanged in all four sets of experimental mice from the density observed in control mice. Instead, the superior function of tumor blood vessels in control mice was due to enhanced development and maturation of the vascular cell populations, leading to increased vessel diameter, greater vessel patency, and reduced vessel leakiness. Tumor vessels in Tks5 knockdown tumors and in tumors in collagen VI null, germline NG2 null, and Mac-NG2ko mice were more poorly functional in each of these respects than tumor vessels in control mice. This suggests that vessel abundance/density may be less important for tumor growth than the details of vessel structure and function [5]. This suggestion is supported by the finding that the extent of perivascular VEGF localization to tumor blood vessels in human oligodendrogliomas correlates with tumor grade but not with vascular density [50]. Accordingly, future studies that focus on mechanisms by which basement membrane-pericyte-endothelial cell interactions contribute to vessel functionality are likely to be more valuable than studies focused on vessel density.

## Figures and Tables

**Table 1:** Comparisons of tumor vessel structure and function.

	1. Collagen VI Null (Brain)	2. Tks5 Knockdown (Mammary)	3. NG2 Null (Mammary)	4. Mac-NG2ko (Brain)
decreased tumor growth	2-fold	4-fold	2-fold	6-fold
decreased vessel density	no change	no change	no change	no change
decreased PC/EC sheathing	no change	ND	1.6-fold	2.5-fold
decreased basement membrane	2-fold	ND	1.5-fold	8-fold
decreased PC maturation	2-fold	ND	2.1-fold	5-fold
decreased EC sprouting	2-fold	ND	2.3-fold	3-fold
decreased vessel diameter	1.5-fold	3-fold	1.4-fold	2.3-fold
increased vessel leakage	3-fold	2.4-fold	3.4-fold	5-fold
decreased vessel patency	1.4-fold	ND	ND	2-fold
increased tumor hypoxia	7-fold	3.3-fold	2.3-fold	12-fold
increased total VEGF-A	1.3-fold	ND	1.5-fold	3-fold
decreased vessel VEGF-A	2-fold	3-fold	ND	3-fold
increased diffuse VEGF-A	2-fold	ND	2-fold	4-fold

Vascular parameters were compared in 4 different tumor models. (1) Germline collagen VI ablation in host stroma of intracranial B16F10 melanomas. (2) Tks5 knockdown in MDA-MB-231 mammary tumors. (3) Germline NG2 ablation in host stroma of MMTV-PyMT mammary tumors. (4) Myeloid-specific ablation of NG2 in host stroma of intracranial B16F10 melanomas. Changes in each parameter are expressed as fold change in tumors in the experimental models compared to tumors in control mice. PC = pericyte. EC = endothelial cell. PC/EC = pericyte ensheathment of endothelial cells. ND = not determined.
